# Making sense of professional enablers’ involvement in laundering organized crime proceeds and of their regulation

**DOI:** 10.1007/s12117-020-09401-y

**Published:** 2020-11-26

**Authors:** Michael Levi

**Affiliations:** School of Social Sciences, Cardiff University, Glamorgan Building, King Edward VII Avenue, Cardiff, Wales CF10 3WT UK

**Keywords:** Money laundering, Organized crime, Enablers, Lawyers, Legal professionals, Effectiveness

## Abstract

Money laundering has ascended the enforcement and criminological agenda in the course of this century, and has been accompanied by an increased focus on legal professionals as ‘enablers’ of crime. This article explores the dynamics of this enforcement, media and political agenda, and how the legal profession has responded in the UK and elsewhere, within the context of ignoring the difficulties of judging the effectiveness of anti money laundering. It concludes that legal responses are a function of their lobbying power, the determination of governments to clamp down on the toxic impacts of legal structures, and different legal cultures. However, it remains unclear what the effects on the levels and organization of serious crimes for gain are of controls on the professions.

## Introduction

Why should we be interested *analytically* in the financial flows and in the professionals and financiers connected with organized crime? There has been intermittent interest since the 1960s and indeed, even centuries earlier, in the “depth of field” of organized crime. ‘The fence’ has been an enduring factual and fictional theme for centuries, for example in Dickens’ *Oliver Twist*, and it is sometimes said that without receivers of stolen goods, there would be less crime.^1^ Yet receivers and the conceptual Stolen Property System have attracted relatively little interest from criminologists or from strategic policing (for exceptions, see e.g. Chappell and Walsh 1973; Langworthy 1989; Mack 1972; Mack and Kerner 1975; Sutton 2014).

Money laundering has been increasingly mainstreamed in this century as a key component of major crimes for gain: *Trends in Organized Crime* itself has published 500 articles containing money laundering as a key word, beginning in 1995 when the journal began. As of mid-October 2020, Google Ngram shows the rising trend in anglophone books containing the phrases ‘money laundering’ and 'organized crime' over the last century (1920-end 2019) thus:

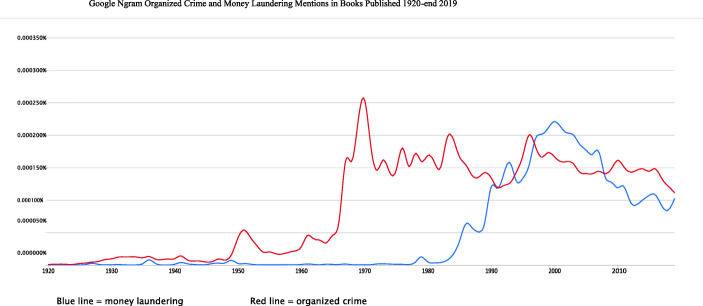



So there appears to be a growing market for (or at least supply of) money laundering texts! Yet despite the immense institutional success of the Financial Action Task Force and the EU in expanding their mandate and legislating the world (Halliday et al. 2020), contemporary investigative journalism is full of examples of ‘under-investigated’ and ‘under-prosecuted’ money laundering rings. As I write, the latest of these is the ‘FinCEN leaks’ which, after a lengthy gestation while some of the financial links were analyzed in the now familiar way across continents, was trailed apocalyptically on September 20, 2020 by the leak’s recipient BuzzFeed as follows (https://www.buzzfeednews.com/article/jasonleopold/fincen-files-financial-scandal-criminal-networks):

The FinCEN Files investigation shows that even after they were prosecuted or fined for financial misconduct, banks such as JPMorgan Chase, HSBC, Standard Chartered, Deutsche Bank, and Bank of New York Mellon continued to move money for suspected criminals.

Suspicious payments flow around the world and into countless industries, from international sports to Hollywood entertainment to luxury real estate to Nobu sushi restaurants. They filter into the companies that make familiar items from people’s lives, from the gas in their car to the granola in their cereal bowl.

The FinCEN Files expose an underlying truth of the modern era: The networks through which dirty money traverse the world have become vital arteries of the global economy. They enable a shadow financial system so wide-ranging and so unchecked that it has become inextricable from the so-called legitimate economy. Banks with household names have helped to make it so.

The international media focus subsequent to these revelations tended to focus on the banks’ ongoing willingness to act for clients they had made reports on, rather than on the lack of public investment in following up the reports they had made, freezing the alleged crime proceeds and prosecuting or taking other action against suspects. One reason for this may be that the inaction of private institutions such as bankers and professionals makes an attractive target for ‘folk devilment’, especially if there is official corruption to complement it. In parallel, alongside a journalistic undercover operation, Al Jazeera was able to shine a light on the Golden Passports scandal in Cyprus, offering Cyprus and therefore EU citizenship for a substantial fee to some less than impeccable non-Europeans, with senior officials allegedly ‘on the take’ and some senior political resignations in the aftermath of publicity^2^; Malta has some very similar publicized issues,^3^ but many other EU countries differ from Cyprus and Malta only as a matter of degree (which is not unimportant): they are less blatant in offering EU-wide passports without much due diligence (Global Witness 2020). The net effect of these revelations in 2020 might be to sharpen the questions about what are the benefits of Anti-Money Laundering (AML) controls and upon whom and what the controls bite (and do not bite) in different parts of the world.^4^

## Organized crime as a target of AML

The regular critiques do not lead properly to the conclusion that money laundering controls have no effect, but there is little research that shows how or to what extent they impact on what crimes and what forms of criminal organization. Organized crime scholars over the past decade have become interested in the ‘scripts’ of organized crime and in the ways in which crimes are organized and shaped by controls: markets vary in the extent to which we know about their extent and shape, however, and given the alleged trillions of dollars involved in money laundering, a valid question is how much of that huge criminal space is well described and understood? Organized crime and money laundering have been put together in a model first developed by the US Presidential (Reagan) Commission on organized crime which reported in 1986. This saw a focus on the money trail as one of the key routes through which its particular forms of organized crime could be combated.^5^

‘If money laundering is the keystone of organized crime, these recommendations can provide the financial community and law enforcement authorities with the tools needed to dislodge that keystone, and thereby to cause irreparable damage to the operations of organized crime’.

*The Cash Connection,* US Presidential Commission on Organized Crime (1986: 63)

This model focusing on the goal of integration or legitimization of the proceeds of crime contains a paradox. If the crime syndicates are planning to finance future crimes, they are definitely not thereby legitimizing the proceeds of past crimes: rather the reverse, since they are both laundering (in the formal legal if not in the analytical sense of cleansing) *and* intending to commit whatever new predicate crime they are planning. This is highlighted to stress an important ambiguity: on the one hand, the dominant cultural image of ‘laundering’ among national, Inter-Governmental Organizations (IMF, World Bank and UN) and Non-Governmental Organizations is indeed the use of sophisticated methods to cleanse ‘dirty money’ (from an ever-increasing range of predicate offences, latterly including foreign bribery and both domestic and foreign tax evasion); but on the other, the *offence* of laundering applies in most jurisdictions to whatever anyone does to hide, transfer or transform the proceeds of any crime, *whether or not this actually legitimizes the funds or is intended to do so*. Some proceeds are moved around to conceal their illegitimate origins in such a way as to defeat a significant financial investigation by competent professionals (though because of resource constraints combined with secrecy havens, this is not likely to happen in practice in many cases). But others are merely simple (self) laundering into accounts in their own or friends’ names, to fund possible future crimes as well as lifestyle expenditures and savings rather than as precursors to integration into the mainstream economy.^6^ Thus, one finds many newspaper reports stating that people have been charged both with drugs trafficking and with money laundering if the police have found a large bundle of cash hidden behind a false wall in their home or buried in the garden or even concealed in their cars.^7^

Many launderers fall in between these extremes. Once he (normally a male) generates a volume of business too large to spend immediately and/or to store physically in a place he considers safe (including a bank account or real estate which may be in the name of others), the drug dealer or other illicit trader will need someone with other skills to launder the revenues, at least on an intermittent if not regular basis if he intends to continue a life of crime. Both will be guilty of money laundering. Though convictions for corrupt financial insiders are rare – as they are also for ‘insiders’ in cybercrimes (Williams et al. 2019) - there can be three parties: the money launderer could be an intermediary who recruits someone inside a financial institution to make the transaction and/or open accounts to facilitate transactions (knowingly or with willful blindness or even naively), or a set of ‘money mules’ with existing accounts recruited (sometimes believing it is a genuine job) to put the crime proceeds through for onward transmission. Nor is it always the case that it is the ‘organized criminal’ who makes the approach to the insider. It can be an insider who is rapacious and seeks out offenders for whom to launder money whether he has always done this or is responding freshly to financial or other pressures. This corresponds to distinction between ‘grass eaters’ and ‘meat eaters’ made by the Knapp Commission (1972) on New York police corruption: grass eaters are those who passively receive bribes, and meat eaters are those who proactively go in search of bribes.

However, there is a constraint which results from an enforcement and regulatory focus: especially if they are going to launder money regularly, insiders within financial institutions need to neutralize internal vigilance by Compliance and/or Money Laundering Reporting Officers, who in theory could go to jail as well as receive large corporate fines for having inadequate money laundering controls. So despite the understandable cynicism when we observe big money sums in corporate sanctions for large-scale laundering, it would be very surprising if internal controls *never* had any effects in situational laundering prevention. Whistleblower accounts and criminal cases tell us only about the ‘failures’ to do so (or rather, intentional decisions to bypass controls), often involving kleptocrats and intermediaries in high level corruption, but also involving drug dealers and human traffickers. In the 1MDB case in which the then Prime Minister of Malaysia Najib Razak has been given a 12 year prison sentence in 2020 for funneling part of billions stolen from the country, senior executives at Goldman Sachs found ways to hide the involvement of wealthy entrepreneur Jho Low, by-passing the strong objections of Goldman’s compliance officers, who sought to stop him becoming a private wealth client (Noonan 2020). In that case, as in Deutsche Bank compliance efforts to reduce the bank’s dealings with Donald Trump pre-Presidency (Enrich 2020), the efforts failed. However, in some (we have no idea what proportion of) others, they have succeeded in reducing particular crimes (though not necessarily in reducing ‘crime’ overall). In 2017, HSBC staff became suspicious about the proposed transfer of $500 million by the son of the former President of Angola, reported it to the authorities, and blocked the account. The filing of the suspicious activity report led to an international investigation, the return of the funds, and the imprisonment of the son^8^ (and following ‘Luanda Leaks’, action against his sister Isobel dos Santos, Africa’s richest woman, including freezing all her accounts): though see Engebretsen and de Oliveira (2020).

In the long period since the first US criminalization measures in 1986, one might have expected a strong evidence base on what happens to proceeds of crime. That is far from being the case. Indeed governments and intergovernmental organizations have spent almost nothing on public research on these issues, while regular costly (pre-Covid-19) international meetings service the global fight against this ill-understood phenomenon and to ‘evaluate’ these efforts. (These evaluations are mainly procedurally to date, though this is slowly changing to ‘real world’ assessment, at least in principle - see Levi et al. 2018; Ferwerda and Reuter 2019). Meanwhile, especially this century, what many governments include in the category of ‘financial crime’ expands ever wider: for example, it is difficult to evade taxes without also being a money launderer, though very few are prosecuted unless they are political opponents of authoritarian governments. In this morass of perpetual control activity, it may not be surprising that there is no coherent ‘theory of change’ that explains and/or predicts what levels of financial investigation and asset recovery will have on the volume or forms of laundered money, nor on the separate question of how these interventions will impact on criminal markets under which circumstances. Instead of serious empirical work (whose absence is strongly critiqued by academics from Naylor 1999 to Van Duyne et al. 2019), there is a compelling cross-cultural narrative of ‘follow the money’ established initially by the use of tax evasion charges to jail Al Capone^9^ that has become a law enforcement mantra since the mid-1980s (Halliday et al. 2020).

The evidence on where the money goes and most recently also where the money comes *from* (Levi 2015) has been seen as a route into answering the question of how concentrated organized crime is, how transnational it is, how ‘complicated’ it is (though by what criteria remain unexplicated) and whether there is any evidence to support the common view developed in the US to fit the drugs market and recycled endlessly ever since, that money laundering always goes through the three stages of placement, layering and integration. The integration concept is nicely captured in the themes of *The Godfather* but is also reflected in an earlier era in the analysis by Bell (1953) that organized crime represents a ‘queer ladder of social mobility’ in America. In most cases, and not disregarding the important point about organized criminals in the UK (and likely elsewhere) seeking *local* or at most regional rather than *national* or *global* control (Campana and Varese 2018), how much appetite is there among most launderers or organized criminals in OECD countries to attain political control or to become titans of finance and industry?

The anti-money laundering transnational legal order has been developing rapidly in recent years to incorporate all crimes great and small, and one of the difficulties for this paper (and for the more substantial review by Levi and Soudijn 2020) is how to differentiate the laundering of organized crime funds from other sources of criminal income, which include tax evasion, grand corruption and the financing of terrorism - which can sometimes involve committing ‘organized crime’ offences. For example, it is hard to maintain that the funneling of billions allegedly stolen in the Malaysian 1MDB scandal was not well organized. It is simply that the principal people involved – including the then Malaysian Prime Minister and his entourage, Goldman Sachs and many intermediaries - would not be viewed by many respectable elites or by the police as ‘organized crime actors’. We might extend this boundary problem to the deliberate falsification of data to regulators e.g. ‘diesel-gate’ emissions, primarily by the Volkswagen Group, and also by the now defunct Takata of safety data for its worldwide manufacture of car airbags. Arguably that behavior involved several actors planning how to commit crimes and get away with them over a long period of time for the pursuit of profit and power. Yet many people (if not many readers of *Trends in Organized Crime*) would balk at the idea of labeling senior executives of major corporations as organized criminals and of describing their distribution of profits as organized crime money laundering, though others might complain if we did *not* so label them for their intentional deception (see Levi 2019 for a discussion). Other contexts of elite misconduct are less challenging to our stereotypical differentiation between white-collar and organized crime: e.g. the investigative media exposure of Operation Laundromat and subsequent scandals shows cross-ties between politicians in the former Soviet Union, organized criminals, professional crime enablers and bankers in the Baltic States, and international finance centers including London and New York. Conventional divisions between white-collar and organized crime are challenged also by accusations about the conduct of Kazakhstan-based mining company ENRC, which has been involved in a long running battle with the UK Serious Fraud Office over its transnational bribery investigation which has so far lasted seven years without a decision to prosecute or not (Burgis 2020a, b). Many millions of pounds have been spent on lawyers’ fees in this and similar cases, competently and aggressively defended as is their legal right. This included allegations that the now retired head of white-collar crime at law firm Dechert procured and/or used hacked emails and was complicit in torture (or ‘robust interrogation’) of a former senior lawyer in the UAE, in connection with defending ENRC; there are allegations that potential witnesses were murdered (Beioley 2020a, b). Dechert was also accused by ENRC of passing on information to the SFO about the case against ENRC’s interests, though the Court of Appeal ruled that litigation privilege would apply to documents shared between ENRC and its then legal advisers, and the SFO could not get access to external or internal legal documents (SFO v ENRC [2018]EWCA Civ 2006; Kemp Little 2019). So some ‘elite’ corporate cases can be as murky as stereotypical ‘organized crime cases’, a fact depicted in movies and contemporary television series about whistleblowers and launderers, from *Chinatown* and *The China Syndrome* onwards.

One useful way of thinking about it is to separate out full-time organized criminals; organized crime facilitation via otherwise legitimate or semi-licit legal and accounting professionals; and transport logistics for crime proceeds. How ‘elite’ the facilitators (including lawyers) of organized crime funds are compared with those that facilitate the proceeds of Grand Corruption is largely unknown and not explicitly examined. In some countries, there is a fusion or at least a strong overlap between some politicians and ‘organized crime’ activities as popularly understood. The involvement of these actors can be analyzed as routine activities (a) knowingly (b) unknowingly and (c) hard to tell if a or b: but the nature of these ‘markets for laundering’ and how asymmetric they are is ill understood.

## Professional enablers

Much of the white-collar crime literature indicates how difficult it is to ‘folk devil’ corporate elites and professionals, whether intentionally or simply as a by-product of media coverage or regulatory/criminal justice interventions (e.g. Levi 2009; Lord et al. 2019). It is not clear where and when the first use of this apparently neutral but often pejoratively used term began. However, a report by the World Economic Forum (2012) on *Organised Crime Enablers* contained a section on Money Laundering Enablers.^10^ The National Crime Agency’s (2014: 1) strategy for tackling ‘High End Money Laundering’ somewhat bizarrely states: “For the purposes of this strategy, we are defining “high end” money laundering as the laundering of funds, wittingly or unwittingly, through the UK financial sector and related professional services.” Though the document did distinguish crimes that needed to hide an audit trail from street level crimes, many might have thought that most money laundering met that description of ‘high level’. It plausibly and reasonably added (p.1) that

Although there are many ways to launder money, it is often the professional enabler who holds the key to the kind of complex processes that can provide the necessary anonymity for the criminal. Professionals such as lawyers, trust and company formation agents, investment bankers and accountants are among those at greatest risk of becoming involved, either wittingly or unwittingly.

Thus, lawyers are generically one set of enablers, and the construct was formalized in the UK’s Serious Crime Act 2015 which criminalized membership of organized crime groups, in line with the EU Directive.

In the US, the more common term for enablers is ‘gatekeepers’: readers may judge for themselves whether this is more or less pejorative as a construct, but the American Bar Association- which (like the Australians) has successfully resisted national regulation for AML – appears to embrace it. In 2002, two years after the Law Society for England and Wales created a Money Laundering Task Force, the US created an ABA Task Force on the Gatekeeper Regulation and the Profession whose principal goal appeared to be ‘hands off’ (https://www.americanbar.org/groups/criminal_justice/gatekeeper/):

Our Task Force was created…to examine U.S. government and multilateral efforts to combat international money laundering and the implications of these efforts for lawyers and the profession. The legal profession, as well as certain financial sector professions, have been characterized as the "gatekeepers" to the international financial and business markets.

The mission of the Task Force is to respond to initiatives by the U.S. Department of Justice and other organizations that will impact on the attorney-client relationship in the context of anti-money laundering enforcement. We are reviewing and evaluating ABA policies and rules regarding the ability of attorneys to disclose client activity and information, and developing a position on the Gatekeeper issue. We are developing educational programs for legal professionals and law students, and organizing resource materials to allow lawyers to comply with their anti-money laundering responsibilities. The goal is to preserve the integrity of the attorney-client relationship.

This is not the place for a detailed legal discussion of how different legal regimes have responded to demands from the Financial Action Task Force to control their gatekeeping function (see Levi 2020). Suffice it to say that the answers lie in political lobbying strengths and in different legal cultures struggling against pressures from the Financial Action Task Force and the European Union, within a context of massive general ignorance of both money laundering and the role of professionals, which does not appear to have gripped the public or politicians in the same way that some forms of street and household crime have. Dealing with kleptocracies has occasionally stimulated high level international action plans (e.g. the Cameron Summit of 2016 - https://www.gov.uk/government/speeches/anti-corruption-summit-2016-pms-closing-remarks), and in the UK, the BBC television series (but not the decade earlier book) *McMafia* stimulated greater political support for enquiries into Russian money of questionable provenance and its impact in Britain. In parallel, the proselytizing by mega-rich former fund manager Bill Browder has led the US, Canada and the UK to enact Magnitsky sanctions against what they politically deem Human Rights abuses, in memory of the former lawyer to a hedge fund who was allegedly killed in a Russian prison at the instigation of the Putin regime, and the Panama Papers and other campaigns have highlighted the overseas assets of Putin and some other prominent politicians (Belton 2020). However, bankers and governments rather than lawyers have been the primary focus of these campaigns. Nevertheless, in October 2020, The German Koln prosecutors issued international arrest warrants for Jürgen Mossack and Ramón Fonseca to answer accusations of forming a criminal organization and complicity in tax evasion in Germany: they are also being sought by the US and are ‘under investigation’ in Panama, which does not extradite its citizens, so they are only prosecutable if they travel abroad.

Nougayrede (2019) has explicated the different cultural factors underlying lawyer regulation in France, the UK and the US, to which I would add that in the UK, the cultural tradition of less legally formalized public-private partnerships has been a strong feature of the lawyer regime as well as other aspects of anti-money laundering (see also Vogel and Maillart 2020). American and Australian lawyers have generated a minimalist approach to their legal obligations to analyze the riskiness of their clients and to report to national Financial Intelligence Units any suspicions about their transactions, while UK lawyers are at the other end of that international spectrum, reporting suspicions of their clients’ transactions more often than the rest of the EU together, despite theoretically having the same regulatory obligations. Notwithstanding that, criticisms of ‘under-reporting’ have continued to be made by senior members of the UK government, the National Crime Agency and NGOs over the past few years, leading to complaints by the legal and other professions that they have been unreasonably stigmatized (see e.g. Cross 2018; Walters 2016), as they have also been for actively defending Legal Aid and immigration issues, and as judges have been for making rulings supportive of Human Rights and critical of some Brexit legislation.

There are multiple ways that this issue can be examined. One is to focus solely on the legal, organizational and political responses to control. The second is to start with what we know about lawyer participation in money laundering and crime (what the UK National Crime Agency (NCA), 2014, refers to as a strategic ‘knowledge gap’) and to consider how we might fill the gaps with reasonably valid knowledge rather than either (a) cynical assumptions about lawyer participation or (b) (by some legal professionals) naïve or faux naïf denials accompanied by demands for proof. Some aspects of the evidence are dealt with in Levi and Soudijn (2020). For our present purpose in focusing only on laundering, we should set aside cases of alleged serious lawyer misconduct in assisting mostly legitimate corporations, because though it may help with an alleged corrupt or fraud scheme, this is merely a precursor to the laundering phase. A lawyer or a sophisticated businessperson can probably launder the proceeds of their own crimes without recourse to a third party ‘enabler’. Someone getting a non-trivial amount of cash from drugs or other illicit services might spend some of it on having fun, and build and/or buy homes for himself and his family – in either his country of origin or locally – with it. It depends on his or her savings ratio and income from crime. If self-laundering is not practical, the laundering can be outsourced to so-called professional money launderers (PMLs) who are contracted by the criminal to solve particular logistical bottlenecks (Benson 2020, Kleemans et al. 2002; Kruisbergen et al. 2014; May and Bhardwa 2018). A PML is a necessity if the offenders want to be able to develop crimes at scale, distinguishable from usually low-skilled and readily substitutable ‘front men’ or ‘straw men’, who might be nominees on property deeds or company documents but who do not plan or execute a laundering scheme.

So the need for enablers depends on the scale and form of the profits from crime, and the seriousness with which the offenders want to conceal them. The classic nail bar, sun-tanning or massage parlour may not require an elaborate corporate construction, though it will require an accountant (who may not need to be a member of a professional body) and a lawyer for property title and transfers. An international business normally would require a lawyer/notary, though not necessarily a complicit one. A 2019 civil confiscation order for £6 million targeted the network around the winner of millions in the UK National Lottery, where it appeared that this offered a partial cover for a web of illicit funds from frauds and from Russia, involving a multi-millionaire businessman, a barrister, and others.^11^ Once the transactions grew larger and more frequent, the lottery win should not have disarmed the banks and professionals, though it apparently did. Middleton and Levi (2005, 2015) examine cases of British lawyers who launder the proceeds of their own crimes such as fraud, but also the smaller number identified of those who launder the proceeds of other people’s crimes, perhaps after mutual attraction through vice and/or blackmail pressure. Changes in ethical legal culture, financial pressures from deterioration in levels of business, and the ownership of law firms may increase money laundering opportunities and ‘needs’ of the firm and/or the individual to launder. Benson (2020) analyzed 20 British cases between 2002 and 2013 in which lawyers or accountants were convicted of money laundering. The cases varied by the purpose of the transactions, the level of financial benefit gained by the professional, and the nature of their relationship with the predicate offender. Whereas acting in the purchase or sale of residential property and moving money through their firm’s client account were the most common means by which lawyers in the cases were involved with criminal funds, there were also lawyers who had: written to a bank to try to get them to unfreeze an account; paid bail for a client using what was considered to be the proceeds of crime; transferred ownership of hotels belonging to a client; written a series of profit and loss figures on the back of a letter; and witnessed an email, allowed the use of headed stationery and provided legal advice for a mortgage fraudster. Four lawyers were knowingly and intentionally involved, but in the majority of cases, Benson concluded that there was no evidence of a deliberate decision to offend or dishonesty on the part of the lawyer (which in my view did not mean always that there was none, but that none could be proven). Although such behaviour did not show that the lawyers were part of the ‘crime group’, their contribution to the goal of the crimes – successful extraction of proceeds – was important.

An American research study examined 123 case files of defendants who had been convicted and sentenced in 2009 in the US Second Circuit on federal money laundering charges (Cummings and Stepnowsky 2011). It noted that 98.4% of convictions were obtained through a plea, and found that ‘lawyers facilitated money laundering, both wittingly and unwittingly, in 25 % of the cases examined’. Of the 10 cases pertaining to lawyers in the final data set of 40 cases, four involved ‘lawyer self-directed schemes’ where the lawyer had committed fraud or embezzlement, and then had laundered his/her own illicit gains. This left only six cases where lawyers were unwittingly involved in facilitating money laundering, five of which pertained to real estate transactions. Cummings and Stepnowsky (2011) concluded that there was no demonstrable evidence to support government proposals that lawyers act as agents of the government by filing suspicious activity reports (SARS) with a federal entity, since lawyers who deliberately launder their own illicit monies will not report suspicious transactions, and any suspicious reporting regime could only possibly be of value in cases where lawyers unwittingly facilitate money laundering! (Though if they were truly unwitting, why would the lawyer have made a SAR? Presumably via education to raise their consciousness.) In that study, only 6 out of 123 cases had lawyers who were unwitting dupes of money laundering, and more than 80% of these cases concerned real estate money laundering. But of course, this may do little more than demonstrate that attorney-client privacy and legal professional privilege make convicting or even prosecuting crooked lawyers too much of a challenge except where they run the scams themselves and in essence are self-launderers!

## Discussion and conclusions

Where offenders and would-be offenders expect lawyers and other professionals to be what Capone called ‘the legitimate rackets’, a plausible hypothesis is that they will make requests to assist in laundering more often, especially but by no means only if the professionals have vices for which they can be blackmailed. Conversely, if criminals think that lawyers are ethical and/or have nothing to blackmail or pressurise them with, fewer requests will be made. These positions may be too binary to match the reality within and between jurisdictions: for example, what are the effects of contraction of legitimate business on lawyers’ suspiciousness of new business and whether conduct fits money laundering typologies? Such ‘differential association’ models are difficult to test, and the data are too weak and anecdotal to enable general inferences to be made. But there is no reason why the involvement of professionals in money laundering should be constant over place and time, and despite the high rhetoric about their flexibility and globalisation, the ease with which offenders who are not members of social elite circles can and do use lawyers in less regulated jurisdictions is largely unexamined, at least publicly. (It fits with the incorrect assertion that offenders who are frustrated in one jurisdiction can simply move their criminal operations elsewhere.) Moreover, it is important not to overstate the involvement of legal professionals in *all* forms of ‘organised crime’: Antonopoulos et al. (2019) have thrown a light on money movement in counterfeit goods, which usually requires little lawyer engagement. Nevertheless, even excluding the misconduct of otherwise legitimate corporations, many higher level offenders do use corporate and trust vehicles to transfer assets and money, especially in frauds; and they use lawyers or notaries when purchasing and selling homes and businesses.

Unlike many areas of transnational organised crime, technologies have played only a minor role in this account. Hacking (as in the data compromise of professionals’ files at Mossack Fonseca in the Panama Papers); leaking (as in the FinCEN files); automated searches for background checks/‘adverse media’ on clients and whether they are on international lists of sanctioned individuals and corporations; technologies of data analysis and cross-matching by journalists such as the International Consortium of Investigative Journalists (all cases) have played their growing part in the defamation-avoiding exposure of matters normally held within what Van De Bunt (2010) evocatively described as the ‘walls of secrecy and silence’. (See also Morselli and Giguere 2006.) But in the FinCEN and some other whistleblowing exposes, only electronic funds and corporate vehicles moved, though sometimes clients and professionals giving legally privileged advice did move as they went physically or electronically around the globe in search of increased trade in ‘invisible earnings’. Data are not kept on how many cases lawyers turn down clients or accept deals that (reasonably or not) may be viewed as legally questionable in retrospect: but journalists for the Organized Crime and Corruption Reporting Project (https://www.occrp.org/en/) and Murray (2015, 2020) have illustrated how lawyers, accountants and corporate vehicles are used by East European and Scottish criminals to develop their economic and social power.

Much attention has been paid to the symbolic struggle of getting lawyers to report suspicions outside of the representation of their clients in legal matters, so the attempts to legislate this become a goal in themselves, generating serious political conflicts in countries such as Switzerland – rowing back reforms that were made to please the Financial Action Task Force (2019 and 2020), and constitutional conflicts in countries such as Canada and some European jurisdictions where the compromise is for lawyers to report first or even only to their local Bar Associations (Levi 2020; Nougayrede 2019; Vogel and Maillart 2020). By contrast with inactivity of lawyer regulation in many countries, in the aftermath of criticisms by FATF in its Mutual Evaluation Report 2020, the UAE suspended the license to practice of 200 law firms for a month until they got into compliance with their systems.^12^ However, some might argue that this was a largely symbolic gesture of 'show-and-tell' compliance, and many transactions of concern are undertaken by firms that tick all the compliance boxes.

The UK situation suggests that unless there is a very marked uptick in investigative resources, many reports from lawyers and others will not receive more than cursory attention from financial investigators and law enforcement unless the clients are already under suspicion, and would not have done so over the past decades (Levi and Gelemerova 2020): this may be true elsewhere, except perhaps in jurisdictions such as Switzerland where more pre-reporting vetting occurs.^13^ In 2018–19, there were 478,437 Suspicious Activity Reports in the UK, including 2774 reports from independent legal professionals, though data on how many *firms* report (or do not report) suspicions are secret, and such data are anonymous, highly classified and relate to *individual* reports and sectors unless they are escalated in rare cases to the UK public-private partnership – the Joint Money Laundering Intelligence Task Force, On the other hand, at a normative level, imposing on lawyers an obligation to consider the legality (even to some, legitimacy) of sources of funds and wealth, and the rationale for the legal constructions they put into effect does not seem in principle to be wrong. If no-one other than the lawyer knows about it and the client is able to do what s/he wishes or find another more willing professional if one turns them down, this is little deterrence or prevention. The extension of overseas tax evasion and corruption as a predicate offence is a potential game changer in the volume of crimes that touch upon the work of the transaction lawyer, which is one reason for the resistance in the US and some other legal professions to onerous Customer Due Diligence rules.

However, no jurisdiction (or the Financial Action Task Force or the European Union collectively) has grappled seriously with the problem of how to judge effectiveness in the regulation of enablers, beyond the massive reduction in ‘crime’ that has not yet happened and which, if serious organised crime did fall (with whatever measurement disputes there might be), they might then make a heuristic ‘causal’ connection to ‘denial of laundering opportunities’. In the absence of a ‘solution’ to the effectiveness of lawyer regulation, there has been a focus on the number of SARs by lawyers and on prosecutions/regulatory interventions against lawyers which, in the eyes of NGOs fighting kleptocracy and the FATF, is never ‘enough’. Like bankers, lawyers are unpopular and especially at times of high social inequality and economic crisis, they make good targets for folk devilment, and there is no reason to think that the struggle for expansion of the rules to include the legal profession will cease any year soon.

## Footnotes

1 It is also the case without clients for illicit goods, there will be less crime. There have been Jews (including ultra-Orthodox Jews) involved in money laundering, but (perhaps partly because of its newness in criminalisation), money laundering has not been a central trope in the anti-Semitism that has been present for centuries in portrayals of Jewish bankers and moneylenders (or Dickens’ Fagin/Shakespeare’s Shylock).

2 https://www.aljazeera.com/news/2020/10/15/cyprus-house-speaker-resigns-following-al-jazeera-investigation; https://timesofmalta.com/articles/view/eu-to-launch-legal-action-over-cyprus-malta-golden-passports.825802.

3 Even including the alleged involvement of the then Prime Minister (who had to resign) and his (arrested) chief of staff in covering up the networks involved in the killing of the investigative journalist Daphne Caruana Galizia (‘Malta Murder Investigation Closes In on ‘Mafia State” https://www.nytimes.com/2019/12/19/world/europe/malta-murder-daphne-caruana-galizia.html?action=click&module=RelatedLinks&pgtype=Article; https://www.theguardian.com/world/ng-interactive/2020/oct/15/justice-on-trial-three-years-after-murder-daphne-caruana-galizia.

4 Increasingly, we have authoritarian regimes using banks’ obligations to report ‘suspicions’ of crime and terrorism to require them to divulge financial transfers by political opponents of the regime - e.g. ‘Banks in Hong Kong advised to report security law breaches to police’ https://www.ft.com/content/4f52cb9c-b069-4b6c-9ef6-f980946f6eb3, 20 October 2020 – or to ‘de-risk’ them as clients. One technique of control is to require foreign institutions and charities to register and be monitored more intensively.

5 See Jacobs (2020) for a recent – almost triumphalist - review of this conventional conception of organized crime in the US.

6 Some countries struggle more than others with this criminalisation process, which is usually done as a reaction to or in anticipation of an evaluation by the Financial Action Task Force of one of its regional groupings. The Netherlands, for example, has shifted its legal position on self-laundering on several occasions.

7 E.g. https://www.dailymail.co.uk/news/article-3307285/Magistrate-58-jailed-burying-270-000-drug-dealer-son-law-s-ill-gotten-gains-garden.html; https://www.independent.ie/irish-news/courts/home-with-bulletproof-windows-sauna-and-72k-buried-in-garden-proceeds-of-crime-high-court-rules-37724357.html.

8 https://www.complianceweek.com/opinion/hat-tip-to-hsbc-for-stopping-money-laundering-saving-lives/29324.article.

9 If only Capone had paid his taxes on his illicit income! It is an open question to what extent contemporary criminals do declare all their income from crime and pay tax on it – a claim often made but seldom if ever tested. If they do not declare all their illicit income to the revenue agencies, they have not fully legitimized it.

10 Full disclosure: the author was a member of the report drafting team.

11 https://www.thetimes.co.uk/article/lottery-winner-hit-with-6m-bill-over-money-laundering-h6sbdc0dl. The NCA uncovered an international money laundering network that saw hundreds of millions of pounds transferred through more than 100 bank accounts held globally including in the UK, Russia, Hong Kong, and Switzerland. The lottery winner’s husband’s activities first came to light during another NCA civil recovery investigation involving convicted drug smuggler Amir Azam where £4million in assets were recovered. He structured his wealth so it was in his wife’s name; they rented a London flat in Belgravia costing £2000 a week. The couple hired a private jet for a foreign trip, holidayed in Cannes and Dubai, shopped at Harrods and built a swimming pool at their country home. So not all was saved and integrated! See https://nationalcrimeagency.gov.uk/news/eight-year-nca-investigation-results-in-multi-million-pound-asset-recovery-including-luxury-hotel-and-100k-bentley (accessed 12 May 2019). In another case, following *civil* proceedings and an Unexplained Wealth Order, the unprosecuted head of the 88 M business group gave up to the NCA 45 properties and four parcels of land in London, Leeds and Cheshire, plus £584,000 in cash that was the subject of an account freezing order. Some accountants and lawyers who worked with him or his businesses have been reported to their professional regulators, with outcomes yet unknown. His business front made it not implausible that the money came from licit activities (*The Times,* 7 October, 2020).

12 They failed to appoint a compliance officer, fill out a questionnaire relating to money-laundering laws or update their data as requested. https://fincrime.report/news/2020/10/23/uae-suspends-licenses-of-200-law-firms-for-breaching-aml-regulations/ (Accessed 25 October 2020).

13 Though it would be incorrect to think that lawyers in the UK do not consider carefully before they make Suspicious Activity Reports on their clients, even if some of them are made defensively to avoid the risk of money laundering charges. Lawyers in European jurisdictions may have to drop their clients if they make a SAR on them, so the *angst* can be considerable: they report far more rarely (Levi 2020).
